# Biology of phosphatidylserine (PS): basic physiology and implications in immunology, infectious disease, and cancer

**DOI:** 10.1186/s12964-020-00543-8

**Published:** 2020-03-11

**Authors:** David C. Calianese, Raymond B. Birge

**Affiliations:** Rutgers University, The State University of New Jersey, Newark, New Jersey USA

## Abstract

Phosphatidylserine (PS) is an anionic phospholipid found on the membranes of a variety of organelles throughout the cell, most notably the plasma membrane. Under homeostatic conditions, PS is typically restricted to the inner leaflet of the plasma membrane. However, during cellular activation and/or induction of cell death, PS is externalized on the outer surface via the activation of phospholipid scramblases. Externalized PS not only changes the biochemical and biophysical properties of the plasma membrane but also initiates a series of interactions between endogenous extracellular proteins as well as receptors on neighboring cells to stimulate engulfment (efferocytosis) that influence the surrounding immune milieu. In this thematic series published in *Cell Communication and Signaling*, we feature review articles that highlight recent work in the field of PS biology, including the biochemistry and physiological significance of PS externalization, therapeutic applications and efforts to target PS, as well as posit open questions that remain in the field.

## Introduction

Higher eukaryotic cells and multicellular organisms have evolved efficient immune regulatory mechanisms in order to recognize ‘self’ from ‘non-self’. These mechanisms function at both the innate and adaptive arms of the immune system and involve a series of pattern recognition motifs as well as other cell surface determinants to relay signals via Pathogen recognition receptors (PRRs) such as Toll-like receptors (TLRs) in order to initiate pro-inflammatory, immune activating cellular outcomes, leading to pathogen clearance.

Concomitantly, higher eukaryotic cells and multicellular organisms have also evolved important immune regulatory mechanisms to recognize effete dying cells from healthy live cells. Although recognition of effete dying cells is multifactorial and depends on the reorganization of membrane proteins, carbohydrate and sialic acid residues, and lipids, the relocalization of Phosphatidylserine (PS) from the inner surface of the plasma membrane to the outer external surface has emerged as one of the most important and emblematic signals for dying cells to be recognized by phagocytes, as well as to elicit complex immune modulatory signals. PS externalized on dying cell membranes mediates the interactions between the effete cell and its phagocyte through a cohort of receptors that recognize PS directly or indirectly through bridging ligands. These PS receptor interactions ultimately lead to engulfment, a process called *efferocytosis*, and the immunological consequences of these events are generally characterized by the release of immune dampening and inflammatory resolution signals that prevent a systemic auto-immune response in healthy tissues. Additionally, PS dysregulation has been characterized in a variety of diseases, including those intrinsically programmed in the host as well as those extrinsically derived via pathogens, thus further emphasizing the critical nature of PS biology in pathophysiology and infectious disease.

Given the rapid pace of research in the areas of host immune responses, immuno-oncology and pathogenesis of infectious diseases, we are pleased to organize a thematic series in *Cell Communication and Signaling* that is dedicated to summarizing the extant progress on PS research and related signaling pathways in immunology, disease etiology and pathogenesis. When we started this project, our plan was to help those not yet captive of the field to evaluate the impact this work has as well as its historical importance in cancer biology and immunology. We therefore chose to ask scientists whom we consider thoughtful and provocative to reflect on their areas of expertise. In this respect, our contributors have risen to the challenge and have brought forth analysis and observations that enable a more global view of the field while appreciating where it is moving; from a basic biological standpoint to its potential clinical relevance and application.

**Fig. 1:**
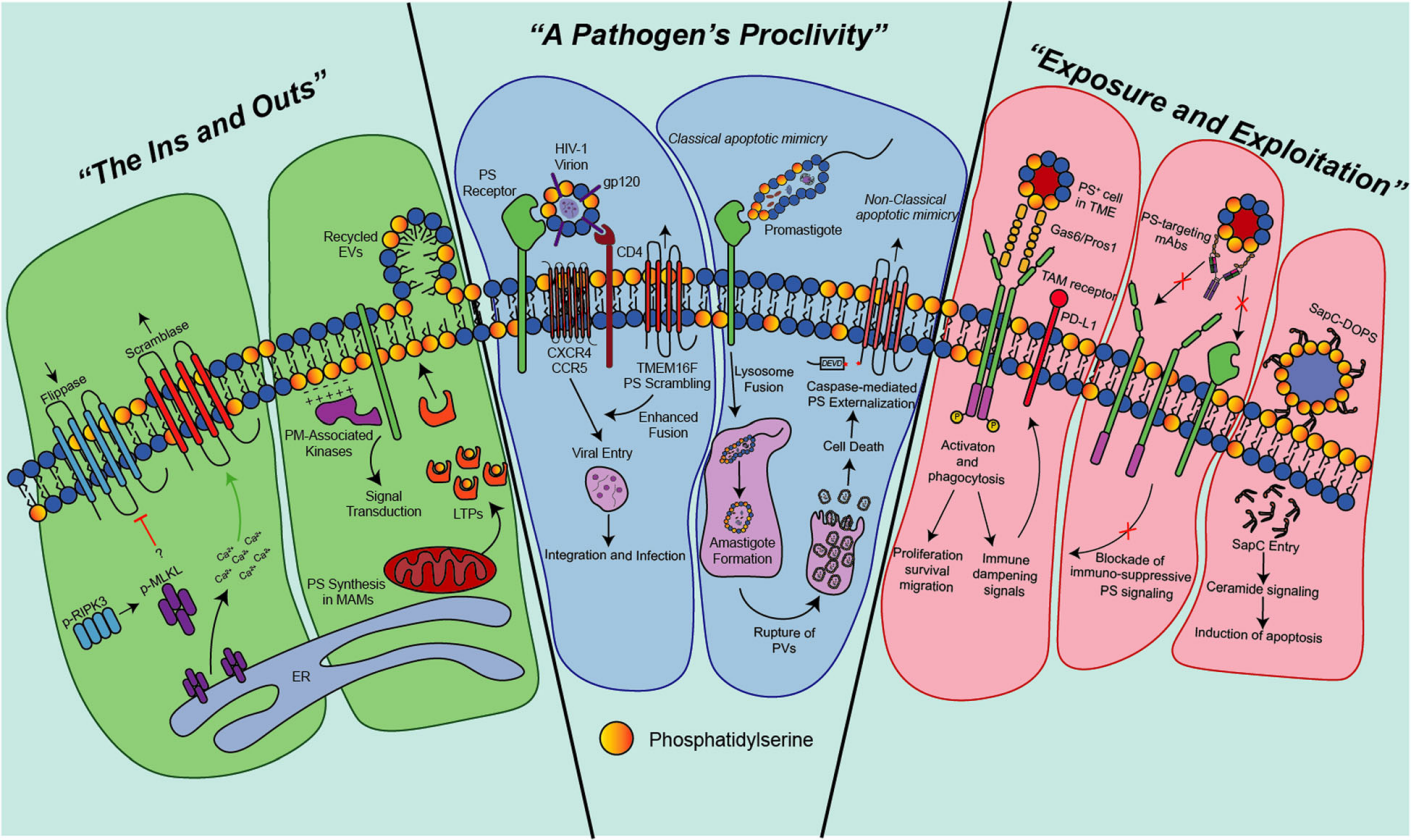
Biology of phosphatidylserine (PS). Included in this figure is a summary of the articles included in this thematic series broken down themes. “*The ins and Outs”:* Shlomovitz et al. describe the externalization of PS in a caspase-independent form of cell death called *Necroptosis*, and the immunological consequences that accompany this process. Kay et al. summarize the synthesis, transport, and intrinsic functions that PS serves within eukaryotic cells. “*A Pathogen’s Proclivity”*: Chua et al. discuss the tendency of virions, notably HIV, to externalize PS on their surface and how apoptotic mimicry influences viral entry and downstream immunological signaling. Wanderley et al. distinguish between *Classical* and *Non-classical* apoptotic mimicry in the context of protozoan infection and disease progression. *“Exposure and Exploitation”*: Burstyn-Cohen et al. characterize the PS-receptor family Tyro3, Axl, Mertk (TAM) as well as its bridging ligands, Gas6 and Pros1, and their implication in immunomodulation in cancer. Dayoub et al. discuss the repertoire of PS-targeting mAbs that are in pre-clinical and clinical development to block pathogenic PS signaling and stimulate an anti-tumor response. N’Guessan et al. describe a novel PS targeting modality that involves Saposin C containing DOPS vesicles, which target to PS externalizing cells in cancer in order to initiate a ceramide-mediated cell death program

The reviews presented in this special issue are organized into three general themes to emphasize how the PS field has progressed in recent years (Fig. 1). These include (i) mechanisms of PS transport and externalization. (ii) PS exposure and apoptotic mimicry by viruses and protists, and (iii) targeting of PS for therapeutic utility.

### Mechanisms of PS transport and externalization: “The ins and outs”

In the first theme, we feature reviews that describe PS synthesis, dynamics, and membrane trafficking, as well as how these processes are compromised in cell death. In the review by Kay and Fairn [1] authors describes the topology, localization and intracellular function of PS, focusing on the synthesis, intracellular transportation to organelles, as well as the biochemical and biophysical properties of PS for membrane structure and function. Authors describe genetic tools used to identify subcellular localizations and concentrations of PS, and subsequently summarize current studies on how PS is transported to the plasma membranes, highlighting methods used for the identification of PS concentrations in membrane microdomains. Finally, the authors address PS in a biochemical context, that PS provides a critical charge density across the inner plasma membrane to interact with polycationic regions of proteins that regulate pivotal cell signaling cascades, caveolae function, endocytosis and recycling.

In a second review by Shlomovitz, Speir, and Gerlic [2], authors describe an underappreciated area of PS biology in comparing PS externalization and its immunological consequences during caspase-independent cell death (mainly necroptosis). The process by which PS is externalized during apoptosis and the immunologically “silent” consequences that follow have been studied at length; here authors provide a perspective of this process in caspase independent forms of cell death which are associated with outcomes that are distinct from that of apoptosis. This work contains a description of basic parameters of apoptosis, necrosis, and necroptosis, and subsequently summarizes current evidence where PS is externalized under these different conditions. The authors discuss the idea that unique signaling cascades are involved in PS externalization during apoptosis and necroptosis, that may depend on factors such as caspase activity, intracellular levels of Ca^2+^, Cl^−^ anion efflux, and differential regulation of flippases and scramblases. The presence of PS externalizing apoptotic and necroptotic bodies that are released by dying cells and their potential to regulate the surrounding immune milieu is further detailed. Finally, the authors argue that processing of apoptotic versus necroptotic cargo (not solely PS per se) may influence the immunological consequences of the dying cells within phagocytes. The idea that PS externalization should not be viewed solely as a caspase-dependent signaling represents a recent development in this field, opening up new exciting area of research arguing that not all PS signaling is equivalent.

### PS exposure and apoptotic mimicry by viruses and protists: “A pathogen’s proclivity”

In a second theme, contributed by Chua et al. [3] and Wanderley et al. [4], these authors describe unique ways that viruses and pathogenic protists utilize PS to gain access to target cells and modulate immune responses, a process referred to as *apoptotic mimicry*. In the review by Chua and colleagues, authors provide an interesting and topical review describing the role of PS externalization in the Human Immunodeficiency Virus (HIV) lifecycle with a main focus on PS in the context of infection. Several main aspects of PS function in HIV infection are addressed including: (i) how PS regulates recognition and entry of HIV into CD4-expressing cells (binding, fusion, entry, and replication), (ii) the role of various PS receptors and PS bridging factors such as TIM-1 and Gas6/Pros1 in viral entry, (iii) roles of PS receptors in trapping budding virus, (iv) mechanisms behind PS exposure on the viral envelope, and (v) how PS exposure regulates clearance of dying HIV-infected cells. Authors also expand an emerging field as to what is known about PS externalization and PS receptors with other viruses, such as Ebola and Influenza, and summarize their thesis that PS targeting might by important to interface with antiviral strategies.

In a second review, Wanderley and colleagues elaborate on this theme and describe protist apoptotic mimicry via PS exposure, and how these processes can permit parasitic infections, evade host immune responses, and establish pathologies. The authors distinguish between two major types of apoptotic mimicry: Classical and Non- Classical. In classical apoptotic mimicry, parasites can expose PS directly, whereas in non-classical parasites utilize host PS in order to establish infections and facilitate disease progression. Subsequently, they effectively describe several paradigms for each type of apoptotic mimicry in the context of *Leishmania amazonensis*, *Trypanosoma cruzi*,, and *Plasmodium* infections. Within this review PS externalization and apoptotic mimicry is described in multiple independent parasitic infection strategies and employed as evolutionarily conserved pathogenesis mechanisms.

### Targeting PS for therapeutic utility: “Exposure and exploitation”

Finally, in the third theme, contributed by Burstyn-Cohen et al. [5], Dayoub et al. [6], and N’Guessan et al. [7], these authors converge on a unifying emerging theme that describes current ideas that PS externalization is a potential targeting strategy in cancer biology. In the review by Burstyn-Cohen and Maimon, the authors first describe the interplay between externalized PS on apoptotic and stressed cells in the context of Tyro3, Axl, and Mertk (TAM) receptor activation, a series of homologous receptors that bridge PS via their ligands Protein S (Pros1) and Growth-arrest specific 6 (Gas6). The authors outline basic elements of PS-Gas6/Pros1-TAM signaling, and subsequently summarize current evidence that (i) PS is externalized under both apoptotic and non-apoptotic conditions such as cell stress, (ii) TAM receptors depend on the PS for maximal robust TAM bioactivity, (iii) PS-ligands-TAMs can act both as autocrine factors on tumor cells and as paracrine factors in the tumor microenvironment (TME), and (iv) that PS-TAM signaling regulates multiple aspects of inflammation and immune resolution. Finally, the idea that PS and TAM signaling in cancer can be contextual and the consequences or existence of these signals depend on the specific cancer phenotype, and whether tumors are tolerogenic or inflamed is nicely articulated.

In the article by Dayoub and Brekken, the authors provide an interesting and topical review describing the pre-clinical and clinical developments of a series of therapeutic mAbs that target externalized PS on the surface of stressed tumor vasculature and blockade of two families of cognate receptors, T-cell immunoglobulin and mucin containing (TIM) and TAM, implicated in several cancers. The authors focus on the historical significance of PS as an unanticipated “universal” tumor marker, and then describe expression patterns of TIMs and TAMs and their potential role in immune modulation, such as T and NK cell exhaustion and effects on innate signaling on DCs and Macrophages. The current stage in development of these mAbs and its combinatorial partners is effectively summarized and organized. Targeting PS externalization and blocking its corresponding oncogenic functionality and immune evasive provides a clear rationale for clinical targeting of PS in cancer biology.

Finally, N′ Guessan, Patel, and Qi continue this theme, and describe a novel and promising PS-targeting nanovesicle (SapC-DOPS), comprised of the lysosomal protein Saposin C and dioleylphosphatidylserine. The authors first describe the biochemistry and structural organization of SapC-DOPS vesicles, their potential mechanisms of cell killing via ceramide signaling, and current evidence that particles specifically target externalized PS on tumor cells but not naïve cells. Various in vivo and orthotopic models are described in this review whereby SapC-DOPS has been reported to selectively target tumors, including intracranial tumors that target across the blood brain barrier, and how these particles may be used with other combinations that increase exposed PS (ie. fractional radiation) to enhance overall therapeutic potential. Finally, comments on the safety profiles in phase I clinical trials are made and the progression of this potentially exciting new therapeutic application.

### Concluding remarks

In recent years, it has become clear that PS externalization on eukaryotic cells, as well as on viruses, bacteria, and protists, have profound biochemical and immunological consequences. Despite these advances, it is also clear that many important questions remain to be addressed. Among these queries are a better elucidation of the kinetics and complex externalization mechanisms by which PS is externalized, including the lifetime of PS on the external membrane as well as characterization of whether specific locals or “ports of exit” of PS exist within the membrane architecture. Similarly, it will be important to determine what specific regulatory pathways exist to control expression and activity of PS scramblases and flippases, as well as whether specific species of PS (acyl length/oxidation status) have distinct activities. Finally, new therapeutic and immunological strategies that target PS are needed to increase the repertoire of PS targeting modalities (Table 1). Together these minireviews, and others invited into this thematic issue, shed light on emerging areas of PS research and reveal a new appreciation for the growing complexity in PS biology.

**Remaining questions textbox**

- Do specific PS species, that differ in modifications such as oxidation state or acyl chain length, have distinct biochemical and biological functions?
- If so, are there species of PS that are specific for certain cell death programs?
- What are the transcriptional, epigenetic, and post translational modifications that regulate scramblase and flippase expression levels and activation states?
- What additional cellular functions are regulated by expression and activation of scramblases?
- PS externalization in caspase-mediated cell death until this point has been deemed irreversible, is this also the case for cell death programs other than apoptosis?
- Is PS externalized on exosomes and other multivesicular bodies distinct from PS externalized on apoptotic cells?
- Do different PS receptors cooperate and function akin to an immunological synapse to stimulate efferocytosis?
- A gain-of-function mutation of PSS1 leading to the overproduction of PS results in severe craniofacial abnormalities. What are the sensors that regulate the levels of PS within healthy cells? Do intracellular levels of PS within the cells fluctuate under certain conditions?
- What is the PS binding proteome, and what intracellular and extracellular signaling pathways are regulated by PS binding proteins?
- Certain viruses, protozoans, along with other pathogens have been characterized to externalize PS on their outer surface in order to gain entry into cells and/or modulate the immune response. What are the species of PS found on their surface? How were they derived?
- Is PS externalized on the surface of activated cells (ie. Immune cells) distinct from PS molecules externalized on apoptotic cells? Are they equivalent in activating PS receptors and downstream signaling cascades?
- Besides PS targeting monoclonal antibodies, what additional strategies can be developed to target PS in cancer and infectious disease?

### Call for papers

Cell Communication and Signaling encourages additional submissions on this research topic. If you believe that you can add to one or more the questions above, please to submit your manuscript to become a part of this CCAS thematic series.
